# Utility of thermography for assessing the success and spread of erector spine plane block in video assisted thoracoscopic surgery

**DOI:** 10.3389/fmed.2026.1820245

**Published:** 2026-04-17

**Authors:** Martins Ansons, Manuel Granell, Mara Klibus, Marina Sarkele, Jevgenijs Proskurins, Olegs Sabelnikovs

**Affiliations:** 1 Department of Anaesthesiology, Intensive Care and Clinical Simulations, Riga Stradins University, Riga, Latvia; 2 Department of Anaesthesiology, Pauls Stradins Clinical University Hospital, Riga, Latvia; 3 University of Valencia, Valencia, Spain; 4 Consorcio Hospital General University of Valencia, Valencia, Spain

**Keywords:** erector spinal plane block, infrared thermography, intercostal blockade, sympathetic blockade, video-assited thoracoscopic surgery

## Abstract

**Background:**

Objective assessment of the success and spread of interfascial plane blocks remains challenging. Infrared thermography may offer a non-invasive method to evaluate erector spinae plane block (ESPB) by detecting cutaneous temperature changes related to sympathetic blockade.

**Methods:**

In this prospective observational study, adult patients undergoing video-assisted thoracoscopic surgery were allocated to either general anesthesia alone or ESPB combined with general anesthesia. Infrared thermographic imaging was performed preoperatively and postoperatively. Intraoperative opioid consumption, postoperative pain scores, and time to first strong opioid requirement were recorded.

**Results:**

Thirty-one patients were analyzed. Intraoperative opioid consumption was significantly lower in the ESPB group (*p* < 0.001). Thermography demonstrated localized postoperative temperature changes (T6 and T7); however, these changes were modest in magnitude and did not demonstrate statistically significant between-group differences at individual dermatome levels.

**Conclusion:**

In the ESPB + GA group, infrared thermography detected increased postoperative temperatures localized in dermatomes T6 and T7, consistent with sympathetic effects related to the erector spinae plane block (ESPB) used in VATS; therefore, infrared thermography monitoring can be considered clinically relevant and it can be used to assess the metameric extent achieved by ESPB; in addition, the ESPB + GA group showed a reduction in the need for intraoperative opioids, better postoperative analgesia, and a later need for postoperative opioid rescue.

## Introduction

Incidence of lung cancer in Europe remains high. It is the third most common type of cancer in women and second most common type of cancer in males (1). In Latvia the incidence of lung cancer reaches 105.1 cases per 100,000 in males and 19.2 cases per 100,000 in females (2).

Surgical intervention with resection of the affected portion of the lung remains one of the cornerstones of treatment. Currently the more traditional approach of thoracotomy has been mostly replaced by less invasive methods such as video-assisted thoracoscopies. This method is associated with lower post operative pain scores and faster recovery. Despite this, it is still associated with strong post operative pain and a 7.7% risk for the development of chronic post operative pain (3).

To relieve intra- and post operative pain in patients undergoing VATS a number of peripheral regional anesthetic techniques have been used (4–6). However most of them are volume blocks and as such rely on the spread of local anesthetic between fascial layers, making it difficult to ensure adequate spread and thus sufficient effect (7).

Currently there are no objective methods to assess the success and spread of peripheral nerve blocks, especially the success of interfascial nerve blocks. An area of some recent research in this topic is the use of thermal imaging, especially infrared thermography of the skin for the assessment of regional anesthetic techniques (8–10). It has shown good results in assessing the success of peripheral nerve blocks, which target specific nerves, such as nerve block of the brachial plexus (9). Even, some published reviews suggest the general use of thermography for most locoregional blocks (11, 12). However, some reviews indicate that thermography would only be valid for detecting a significant and reliable increase in temperature in the most distal parts of the body (e.g., fingers and toes) but not in more proximal areas; furthermore, the authors suggest that significant increases in skin temperature will only be observed in patients presenting with initial vasoconstriction (12).

The novel aspect of this study is the use of a noninvasive method—infrared thermography—to assess erector spinal plane block (ESPB) by detecting changes in skin temperature associated with sympathetic block, based on the evaluation of the temperature difference between the blocked side and the unblocked side.

## Methods

The study was performed in accordance with the Declaration of Helsinki. Ethical approval, including an approved extension, was obtained from the local institutional ethics committee, and informed consent was obtained from patients. The study was approved by the Ethics Committee of Riga Stradiņš University (date: 21.10.2024, approval number 2-PĒK-4/643/2024).

Patients undergoing planned video-assisted thoracoscopic surgery were randomly allocated to one of the following groups:

- *ESPB + GA group (Group 1)*: Patients received ultrasound-guided erector spinae plane block at T5 using 30 mL of 0.375% ropivacaine prior to induction of general anesthesia, followed by standardized general anesthesia
- *GA group (Group 2)*: Patients received standardized general anesthesia without ESPB.

### Inclusion and exclusion criteria

#### Inclusion criteria

1. Planned Video-Assisted Thoracoscopic Surgery (VATS) for lung resection.
2. ASA Physical Status Classification Grade I-III.
3. Adults aged 18 years or older.
4. Minimum body weight of 50 kg.
5. Body Mass Index ≤ 30 kg/m^2^.
6. No history of local anesthetic allergy.
7. Signed Informed Consent Form provided by the patient.

#### Exclusion criteria

**Figure 1:**
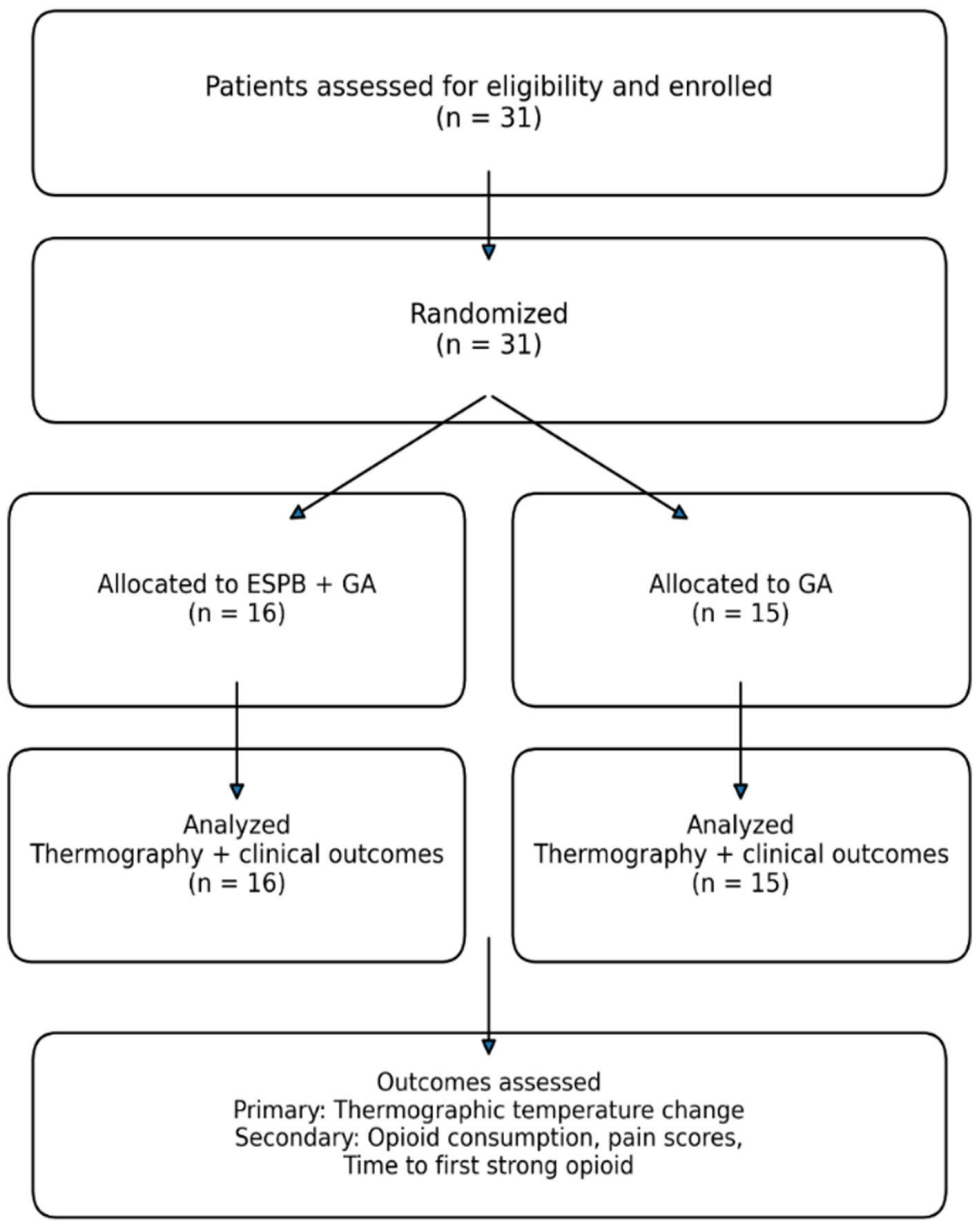
Participant flow diagram. Thirty-one patients were enrolled and randomized to ESPB + GA (*n* = 16) or GA (*n* = 15). All randomized patients completed the study and were included in the final analysis.

1. The patient does not meet the inclusion criteria.
2. The patient has active inflammation in the area where the locoregional block is to be performed.
3. The patient has lesions in the area where the locoregional block is to be performed (Figure 1).

### Procedure

All patients who were included in the study were brought to the operating room prior to the ESPB. Patients were positioned on the operating table with their upper torsos exposed prior to administration of the ESP block. Patients were allowed to acclimatize to the room, which had a constant temperature of 21° C while peripheral venous access was gained and monitoring equipment, which included pulse oximetry, electrocardiography and non-invasive blood pressure cuff was placed.

Thermographic imaging was performed under standardized conditions in both study groups. Baseline intraoperative imaging was obtained 15 min after induction of general anesthesia, once patients were positioned in the lateral decubitus position. The patients were heated using a mattress placed on the operating table set at 37° C and their lower torsos and legs were heated using thermal blankets. Postoperative imaging was performed at the end of surgery under the same positioning and warming conditions. All images were acquired using a FLIR C3 thermal camera positioned at a fixed distance of 80 cm perpendicular to the patient’s back.

For the intervention group we performed ESPB at the level of 5th thoracic vertebrae on the side of the planned surgery using sterile technique and ultrasound guidance using a Mindray TEX20 Ultrasound machine with a L12-3VNs linear transducer which has a bandwidth of 3.0–12.9 MHz. For the block we used 30 mL of 0.375% Ropivacaine (B. Braun) and B. Braun Stimuplex A insulated 22 Gauge needles. Local anesthetic was injected in the fascial space above transverse process of the corresponding vertebrae.

General anesthesia was induced using intravenous Propofol 2–2.5 mg/kg (Baxter) and intravenous fentanyl 3–5 mcg/kg (Kalceks). After induction of general anesthesia, a bolus of intravenous atracurium (Kalceks) of 0.5 mg/kg was given to facilitate the double-lumen tube insertion. After intubation the patient was positioned on their sides to allow for surgical access. General anesthesia was maintained using inhaled sevoflurane (AbbVie) with minimal alveolar concentration value of 1 and intravenous boluses of fentanyl as necessary (Base line MAP/HR increase over 20%). The patients received intravenous bolus of 8 mg of dexamethasone (Unifarma) after the induction of general anesthesia and received 50 mg of intravenous dexketoprofen (Kalceks) and 1 g of intravenous paracetamol (Fresenius Kabi) near the end of the surgery. Before surgical site closure the surgical team performed a visually guided intercostal nerve block using 15 mL of 0.25% bupivacaine in all patients. Intraoperative fentanyl consumption was recorded as an indicator of intraoperative analgesic adequacy associated with surgery under general anesthesia alone or combined with spinal erector plane block. Since visually guided intercostal nerve block was performed at the end of surgery, it did not influence intraoperative opioid requirements. Therefore, intraoperative fentanyl consumption in both groups may reflect the analgesic effect of combined spinal plane block during surgery. Pain at rest was assessed using a visual analog scale (VAS at rest) upon arrival at the ICU, and then at 6, 12, and 24 h postoperatively. In the first 24 h the patients received 1 g of intravenous paracetamol every 6 h and 30 mg of intravenous ketorolac every 8 h. In cases of strong postoperative pain (VAS > 4), the patients received 100 mg of intravenous tramadol (up to 4 times per 24 h). Data regarding opioid consumption in the post operative period was accumulated. Thermography imaging was analyzed using FLIR thermal studios software. Temperature was measured from the 7th cervical dermatome to the 12th thoracic dermatome on both sides before and after the surgery.

### Statistical analysis

Statistical analysis was performed using SPSS version 25 (IBM Corp., Armonk, NY, USA). Descriptive statistics are reported as mean ± standard deviation. Normality of continuous variables was assessed using the Shapiro–Wilk test and visual inspection of histograms.

Between-group comparisons were performed using independent samples *t*-tests. Within-group comparisons were evaluated using paired-samples *t*-tests. Multiple comparisons were performed across multiple dermatomes. Given the exploratory nature of this study and limited sample size, *p*-values are reported descriptively and should be interpreted cautiously. Statistical significance was defined as *p* < 0.05 (two-tailed) (Table 1).

**Table 1:** Demographic and intraoperative data.

Variable	Group	*N*	Mean	Median	SD (Standard Deviation)	SE (Standard Error)	*p*-value
Gender (m/f)	1	16	1.56	2.00	0.512	0.128	0.574
	2	15	1.67	2.00	0.488	0.126	
Operating room time (min)	1	16	222.38	217.50	56.835	14.209	**0.009**	2	15	165.67	170.00	46.401	11.981	0.106
	Surgical time (min)	1	16	151	150	60.9	15.7	
2		15	118	125	48.8	12.6	
Opioid consumption (μg/kg) during surgery	1	16	2.28	2.00	0.515	0.129	**<0.001**
	2	15	4.07	4.00	1.335	0.345	
Weight (kg)	1	16	71.94	72.00	11.869	2.967	0.148
	2	15	78.13	78.00	11.801	3.047	
Age (years)	1	16	63.75	63.50	7.707	1.927	0.123
	2	15	68.29	68.50	7.300	1.951	
Height (cm)	1	16	166.00	165.00	7.789	1.947	0.062
	2	15	170.53	171.00	6.346	1.638	

Bold values indicate statistically significant differences between groups (*p* < 0.05).

## Results

There were no statistically significant differences between groups in gender distribution, weight, age, or height (all *p* > 0.05). Opioid consumption during surgery was significantly lower in the ESPB group compared with the GA group (2.28 ± 0.52 vs. 4.07 ± 1.33 mcg/kg, *p* < 0.001), although there were no significant differences in surgery duration between the two groups. On the other hand, the time spent in the operating room was longer in the ESPB + GA group, due to the performance of the locoregional block in that group before surgery began.

In Table 2, dermatome levels marked with the letter “B” (Block) indicate Group 1 (ESPB group) whereas dermatome levels presented without “B” indicate Group 2 (control group). Statistical analysis using an independent Student’s t-test was performed as a between-group comparison, comparing the ESPB group after the block with the control group, rather than comparing measurements with baseline values (Table 3).

**Table 2:** Temperature difference between groups at each dermatome level.

Dermatome level/measurement point	Test (Student’s *t*)	Statistic (*t* value)	df (degrees of freedom)	*p*-value	Mean difference	SE difference
C7 B post	Student’s *t*	0.0857	20.0	0.933	0.0486	0.567
C8 B post	Student’s *t*	0.4367	20.0	0.667	0.2362	0.541
T1 B post	Student’s *t*	0.7150	20.0	0.483	0.3467	0.485
T2 B post	Student’s *t*	0.8239	20.0	0.420	0.3800	0.461
T3 B post	Student’s *t*	1.2428^a^	20.0	0.228	0.5610	0.451
T4 B post	Student’s *t*	1.6887^a^	20.0	0.107	0.7295	0.432
T5 B post	Student’s *t*	2.0246^a^	20.0	0.056	0.8857	0.437
T 6 B post	Student’s *t*	2.3571	20.0	0.029	1.0267	0.436
T7 B post	Student’s *t*	2.5617	20.0	0.019	1.0305	0.402
T8 B post	Student’s *t*	1.8783	20.0	0.075	0.7933	0.422
T9 B post	Student’s *t*	1.3938	20.0	0.179	0.6381	0.458
T10 B post	Student’s *t*	0.7215	20.0	0.479	0.3486	0.483
T11 B post	Student’s *t*	0.6886	20.0	0.499	0.3371	0.490
T12 B post	Student’s *t*	0.4371	20.0	0.667	0.2124	0.486
C7 post	Student’s *t*	−1.7352	20.0	0.098	−0.9981	0.575
C8 post	Student’s *t*	−1.5363	20.0	0.140	−0.8762	0.570
T1 post	Student’s *t*	−1.5810	20.0	0.130	−0.8571	0.542
T2 post	Student’s *t*	−1.6350	20.0	0.118	−0.8619	0.527
T3 post	Student’s *t*	−1.7312	20.0	0.099	−0.8371	0.484
T4 post	Student’s *t*	−1.9401	20.0	0.067	−0.8848	0.456
T5 post	Student’s *t*	−1.9031	20.0	0.072	−0.8638	0.454
T6 post	Student’s *t*	−1.4876	20.0	0.152	−0.6829	0.459
T7 post	Student’s *t*	−1.0199	20.0	0.320	−0.5248	0.515
T8 post	Student’s *t*	−0.6431	20.0	0.527	−0.3552	0.552
T9 post	Student’s *t*	−0.4246	20.0	0.676	−0.2371	0.559
T10 post	Student’s *t*	−0.5093^a^	20.0	0.616	−0.2914	0.572
T11 post	Student’s *t*	−0.7851^a^	20.0	0.442	−0.4267	0.543
T12 post	Student’s *t*	−0.9568	20.0	0.350	−0.5114	0.534

Ha μ_1_ ≠ μ_2_.

a Levene’s test is significant (*p* < 0.05), suggesting a violation of the assumption of equal variances. ESPB + GA group (Group 1), GA group (Group 2).

**Table 3:** Temperature difference at each dermatome level in ESPB group (Group 1).

Paired samples *t*-test							
Pre-op dermatome level	Post-op dermatome level	Test (Student’s *t*)	Statistic (*t* value)	df (degrees of freedom)	*p*-value	Mean difference	SE difference
C7	C7	Student’s *t*	−0.0564	13.0	0.956	−0.0214	0.380
C8	C8	Student’s *t*	−0.0766	13.0	0.940	−0.0286	0.373
T1	T1	Student’s *t*	0.4664	13.0	0.649	0.1714	0.368
T2	T2	Student’s *t*	0.3034	13.0	0.766	0.1357	0.447
T3	T3	Student’s *t*	0.2379	13.0	0.816	0.1214	0.510
T4	T4	Student’s *t*	0.4718	13.0	0.645	0.2429	0.515
T5	T5	Student’s *t*	0.5904	13.0	0.565	0.3071	0.520
T6	T6	Student’s *t*	0.5618	13.0	0.584	0.2857	0.509
T7	T7	Student’s *t*	0.5656	13.0	0.581	0.2786	0.492
T8	T8	Student’s *t*	1.6504	13.0	0.123	0.6857	0.415
T9	T9	Student’s *t*	1.0223	13.0	0.325	0.5143	0.503
T10	T10	Student’s *t*	1.1845	13.0	0.257	0.6000	0.507
T11	T11	Student’s *t*	1.1584	13.0	0.268	0.5929	0.512
T12	T12	Student’s *t*	1.4434	13.0	0.173	0.7214	0.500

Before and after surgery.

Ha μ_Measure 1 − Measure 2_ ≠ 0.

**Figure 2:**
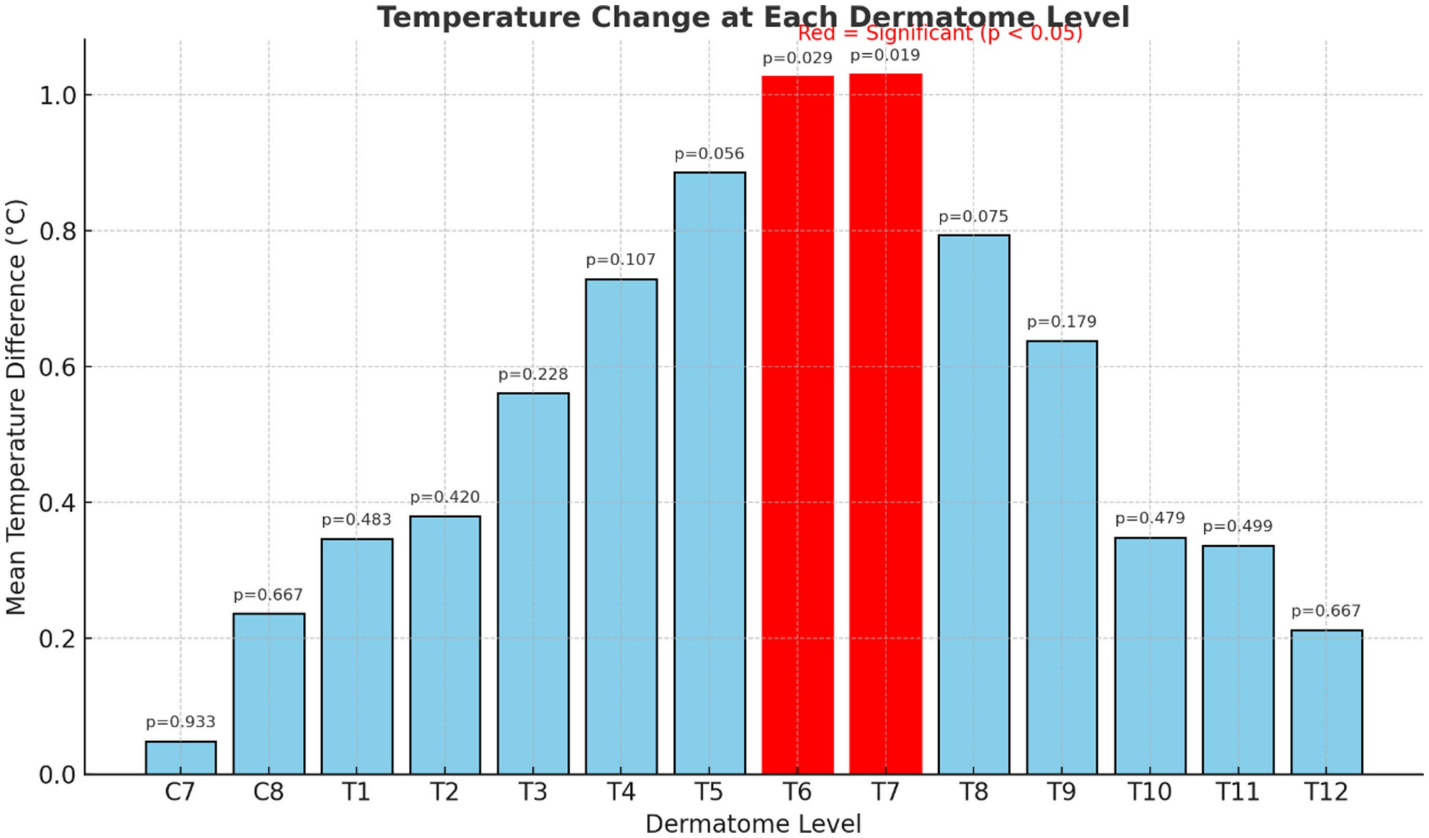
Temperature change at each dermatome level. This analysis refers to a between-group comparison. Specifically, it represents a comparison between groups at each dermatome level. Bars represent mean temperature differences (°C) at the corresponding dermatomes. Highlighted values indicate unadjusted *p*-values < 0.05 and are presented for descriptive purposes only. Although statistically significant changes were observed at T6 and T7 in selected analyses, these findings were not replicated in within-group ESPB pre–post comparisons, and no statistically significant between-group differences were detected at any dermatome level. Therefore, these results should be interpreted as exploratory and descriptive rather than confirmatory.

Significant differences were observed at T6 (*p* = 0.029) and T7 (*p* = 0.019). Levels T5 (*p* = 0.056) and T8 (*p* = 0.075) showed a trend toward significance but did not reach the 0.05 threshold. All other dermatome levels from C7 to T12 did not demonstrate statistically significant differences (*p* > 0.05) (Figure 2).

These findings suggest that, in this subgroup, postoperative changes in cutaneous temperature at individual dermatomes were minimal and did not reach statistical significance (Table 4).

**Table 4:** Temperature difference at each dermatome level in GA group (Group 2).

Paired samples *t*-test							
Pre-op dermatome level	Post-op dermatome level	Test (Student’s *t*)	Statistic (*t* value)	df (degrees of freedom)	*p*-value	Mean difference	SE difference
C7	C7	Student’s *t*	1.91	7.00	0.098	1.30	0.681
C8	C8	Student’s *t*	2.85	7.00	0.025	1.84	0.644
T1	T1	Student’s *t*	3.57	7.00	0.009	2.12	0.595
T2	T2	Student’s *t*	4.07	7.00	0.005	2.30	0.566
T3	T3	Student’s *t*	4.55	7.00	0.003	2.41	0.530
T4	T4	Student’s *t*	4.55	7.00	0.003	2.27	0.500
T5	T5	Student’s *t*	4.59	7.00	0.003	2.29	0.499
T6	T6	Student’s *t*	4.31	7.00	0.004	2.30	0.534
T7	T7	Student’s *t*	3.98	7.00	0.005	2.42	0.610
T8	T8	Student’s *t*	3.90	7.00	0.006	2.56	0.657
T9	T9	Student’s *t*	3.70	7.00	0.008	2.59	0.699
T10	T10	Student’s *t*	3.99	7.00	0.005	2.54	0.636
T11	T11	Student’s *t*	4.38	7.00	0.003	2.51	0.574
T12	T12	Student’s *t*	4.43	7.00	0.003	2.44	0.550

Before and after surgery.

Ha μ_Measure 1 − Measure 2_ ≠ 0.

These results indicate that postoperative changes became significant starting at C8, with a consistent, clinically relevant increase across all thoracic dermatomes (Table 5).

**Table 5:** VAS scores and opioid consumption.

Group descriptives section						
Outcome variable	Group	*N*	Mean	Median	SD (Standard deviation)	SE (Standard error)
VAS 0	1	15	0.00	0.00	0.00	0.000
	2	15	0.400	0.00	0.737	0.190
VAS 6	1	16	2.13	2.00	2.13	0.531
	2	15	4.533	5.00	1.246	0.322
VAS 12	1	16	4.19	4.00	3.15	0.786
	2	15	5.467	6.00	1.356	0.350
VAS 24	1	16	4.25	5.00	1.91	0.479
	2	15	4.467	4.00	0.834	0.215
Time to PO opioids	1	16	8.83	8.00	3.01	0.869
	2	15	6.333	5.00	2.845	0.735

Independent samples *t*-test section						
Outcome variable	Test (Student’s *t*)	Statistic (*t* value)	df (degrees of freedom)	*p*-value	Mean difference	SE difference
VAS 0	Student’s *t*	−2.103^a^	28.0	0.045	−0.400	0.190
VAS 6	Student’s *t*	−3.815	29.0	<0.001	−2.408	0.631
VAS 12	Student’s *t*	−1.452^a^	29.0	0.157	−1.279	0.881
VAS 24	Student’s *t*	−0.404^a^	29.0	0.690	−0.217	0.537
Time PO opioids	Student’s *t*	2.211	25.0	0.036	2.500	1.130

Ha μ_1_ ≠ μ_2._

a Levene’s test is significant (*p* < 0.05), suggesting a violation of the assumption of equal variances. ESPB + GA group (Group 1), GA group (Group 2).

At baseline (VAS 0), pain scores were significantly lower in ESPB group compared with GA group (0.00 ± 0.00 vs. 0.40 ± 0.74, *p* = 0.045). At 6 h postoperatively, ESPB group also reported markedly lower pain scores (2.13 ± 2.13) compared with GA group (4.53 ± 1.25), a difference that was highly significant (*p* < 0.001). At 12 h, pain scores remained numerically lower in ESPB group (4.19 ± 3.15) versus GA group (5.47 ± 1.36), although the difference was not statistically significant (*p* = 0.157). By 24 h, VAS scores were similar between groups (4.25 ± 1.91 vs. 4.47 ± 0.83, *p* = 0.690).

**Figure 3:**
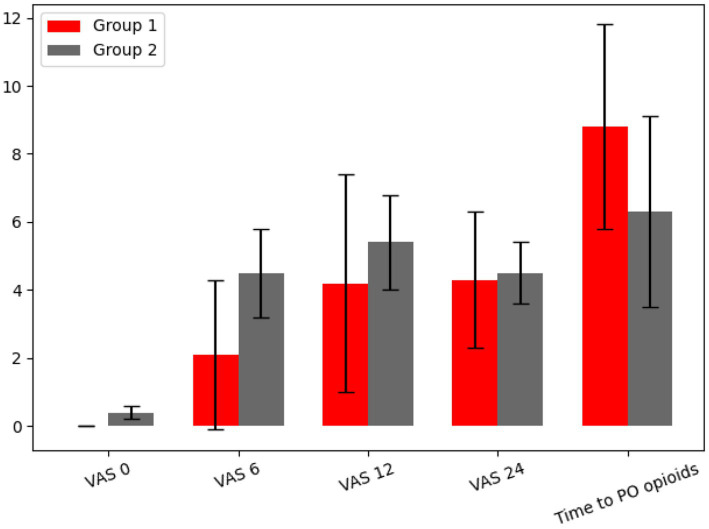
VAS scores at rest (0-10) and time (hours) to first strong postoperative opioid consumption (time to PO opioids). Bars represent mean values with standard deviation error bars for ESPB group and GA group. Significant between-group differences (*p* < 0.05).

The time to first strong opioid analgesia consumption was significantly longer in ESPB group compared with GA group (8.83 ± 3.01 h vs. 6.33 ± 2.85 h, *p* = 0.036). This suggests that ESPB group experienced both reduced early postoperative pain intensity and delayed need for strong opioid medication compared with GA group (Figure 3).

After surgery at baseline (EVA 0) and 6 h after the operation, the ESPB group had significantly lower pain scores compared to the GA group (*p* = 0.045 and *p* < 0.001, respectively). No statistically significant differences were observed at 12 or 24 h postoperatively. Time (hours) to first strong opioid analgesia was also significantly longer in ESPB group (*p* = 0.036).

## Discussion

This study demonstrates that infrared thermography can identify localized postoperative temperature differences at the T6 and T7 dermatomes compatible with possible ESPB-related sympathetic effects after VATS. In addition, patients receiving ESPB + GA showed improved early postoperative analgesia at 0 and 6 h, as well as a delayed need for rescue opioids compared with GA alone. Together, these findings support the role of thermography as an indirect physiological marker of sympathetic blockade rather than a precise method for dermatomal mapping.

Traditionally, the success of regional anesthetic techniques has been assessed using subjective methods such as pinprick or cold sensation testing. These methods require patient input thus are subjective in their nature and often unreliable in practice. Assessing the efficacy of some regional anesthesia techniques is difficult in patients with cognitive impairment, who may benefit from less invasive anesthetic methods. As the use of regional anesthesia continues to expand, the need for more objective assessment tools has become increasingly evident. In this context, infrared thermography has emerged as a noninvasive approach for evaluating block spread, demonstrating promise in peripheral nerve blocks and variable results in interfascial and neuraxial techniques (9–15).

Infrared thermography is based on the detection of long-wave infrared radiation naturally emitted by all objects above absolute zero. At physiological temperatures (30–37 °C), human skin emits predominantly within the 8–12 μm range, allowing accurate, non-contact surface temperature measurement due to its high emissivity. Thermal cameras convert this radiation into calibrated temperature maps (thermograms). However, infrared assessment is limited to superficial tissues, reflecting cutaneous perfusion rather than deeper neural spread, which may partly explain inconsistent findings in thoracic and abdominal regional techniques.

When using regional anesthetic techniques and causing a sensory blockade we most often also inadvertently cause a sympathetic blockade in the corresponding part of the body. When we cause a sympathetic blockade vascular tone in arterioles innervated by the sympathetic nerves is lost, causing vasodilatation and increased blood flow to the peripheral tissues, e.g., skin. This is called core-to-peripheral redistribution and it’s the primary mechanism through which patient’s develop relative hypothermia during anesthesia. This increased blood flow to the skin causes an initial increase in its temperature which can be measured using thermography, later more heat is lost to the environment and the skin temperature decreases, if the patient is under just general anesthesia we would eventually see thermoregulatory vasoconstriction, which would limit the amount of heat lost to the surrounding area due to a only relative loss of sympathetic tone, this does not effectively happen when dealing with regional techniques because nerve signal transmission is blocked and thus heat is continuously lost from the heart to the peripheral tissues. The relatively close proximity of the heart is why we often see contradicting results from thermographic studies examining neuroaxial and peripheral techniques in the thoracic and abdominal regions (16–18).

One possible physiological explanation for the observed thermographic patterns is that patients in the GA group, in the absence of regional sympathetic blockade, may have experienced greater peripheral vasoconstriction, resulting in relatively lower postoperative skin temperature. In contrast, in the ESPB group, persistent sympathetic blockade may have promoted vasodilation and increased cutaneous perfusion on the surgical side, potentially contributing to relative preservation of skin temperature due to core-to-peripheral compartments heat redistribution despite similar environmental exposure (14).

Our study data showed statistically significant temperature changes at the T6 and T7 dermatomes, while no statistically significant differences were observed at other dermatome levels. In this context, the surgical side in the GA group tended to demonstrate relatively lower postoperative skin temperature compared with the ESPB group, which may reflect the absence of regional sympathetic vasodilation. Conversely, in the ESPB group, persistent sympathetic blockade may have promoted increased cutaneous perfusion and relative preservation of skin temperature on the surgical side. This localized pattern may be compatible with the expected distribution of the erector spinae plane block; however, the findings should be interpreted cautiously.

Beyond the physiological changes detected by thermography, clinically meaningful analgesic effects were observed. Baseline demographic characteristics were comparable between groups, with no significant differences in age, gender, weight, or height (all *p* > 0.05). The Although surgical time were no statistically significant differences between groups, intraoperative opioid consumption was significantly reduced in patients receiving ESPB (2.28 ± 0.52 vs. 4.07 ± 1.33 mcg/kg, *p* < 0.001), with opioids in most cases required only to facilitate endotracheal intubation. These findings suggest that ESPB contributed to effective intraoperative analgesia.

Postoperative pain outcomes in our study demonstrated a clear early analgesic advantage in the ESPB group. VAS scores were significantly lower at baseline (0.00 ± 0.00 vs. 0.40 ± 0.74, *p* = 0.045) and at 6 h (2.13 ± 2.13 vs. 4.53 ± 1.25), postoperatively, while differences at 12 h did not reach statistical significance and were no longer apparent at 24 h. Additionally, the time to first postoperative opioid administration was significantly prolonged in the ESPB group (8.83 ± 3.01 h vs. 6.33 ± 2.85 h, *p* = 0.036), indicating delayed analgesic requirement. This pattern—marked early pain reduction with attenuation of intergroup differences over time—is consistent with findings from randomized trials and meta-analyses in thoracic surgery, which report that ESPB significantly reduces pain scores and opioid consumption within the first 6–12 h after VATS, with less pronounced differences at 24 h. Collectively, these data suggest that the principal benefit of ESPB lies in enhancing early postoperative analgesia and reducing immediate opioid requirements when incorporated into multimodal analgesic strategies, including intercostal blockade (19–21).

Our study had some limitations. Firstly, this is a single-center prospective observational study. Secondly, we did not perform neither the skin-prick test nor the cold sensation test to assess the effectiveness of the ESP block, rather we assessed the success of the block based on the lack of response to surgical stimuli. Similar to the approaches reported by Bellantonio et al. (22), Díaz-Bohada et al. (23) and Durey et al. (24), we adopted a more pragmatic evaluation method. Block effectiveness was assessed by the absence of response to surgical stimuli, and temperature changes were monitored using thermography before and after surgery as an indirect indicator of sympathetic and sensory effects. Although this method does not provide detailed dermatomal mapping, it allowed for efficient assessment within our operative workflow.

Thirdly, we did not use a very sophisticated thermal camera, which limited our ability to detect very small temperature changes and better distinguish between adjacent dermatomes.

Another limitation of this study is that postoperative thermography reflects a combination of global thermoregulatory and hemodynamic influences, rather than an isolated regional blockade of the erector spinae plane. Therefore, interference with the results may occur due to the use of active heating devices, lateral positioning, surgical exposure, residual vasodilation related to anesthesia, and systemic medications.

Finally, all our patients were placed in the lateral decubitus position, with the operated hemithorax facing upward, and the lower (non-surgical) hemithorax was warmed using a heated mattress. The lower body was also warmed with a thermal blanket. The use of heating elements is necessary to prevent hypothermia during anesthesia, but it can also affect the thermography results.

In conclusion, Infrared thermography detected localized postoperative temperature differences in T6 and T7 dermatomes, consistent with sympathetic effects related to the erector spinae plane block (ESPB) used in VATS; therefore, infrared thermography monitoring can be considered clinically relevant, as it is a physiological marker of sympathetic block that can be used to assess the metameric extent achieved by ESPB; nevertheless, more multicenter blind studies must be performed in the future to confirm these results; furthermore, the analgesic outcomes in the ESPB + AG group were better than those in the AG group, as the ESPB + AG group showed a reduction in the amount of intraoperative opioids required, better analgesia at 0 and 6 h post-operatively, as well as a delay in the need to administer a dose of post-operative rescue opioid analgesics compared with the GA group.

## Data Availability

The original contributions presented in the study are included in the article/supplementary material, further inquiries can be directed to the corresponding author.
